# Xenotropic and polytropic retrovirus receptor 1 (XPR1) promotes progression of tongue squamous cell carcinoma (TSCC) via activation of NF-κB signaling

**DOI:** 10.1186/s13046-019-1155-6

**Published:** 2019-04-17

**Authors:** Wei-chao Chen, Qiu-li Li, Qimei Pan, Hua-yong Zhang, Xiao-yan Fu, Fan Yao, Jian-ning Wang, An-kui Yang

**Affiliations:** 1 Department of Head and Neck, Sun Yat-sen University Cancer Center, Guangzhou, Guangdong 510060 People’s Republic of China; 2 State Key Laboratory of Oncology in South China, Guangzhou, Guangdong 510060 People’s Republic of China; 3 Collaborative Innovation Center for Cancer Medicine, Guangzhou, Guangdong 510060 People’s Republic of China; 4 Guangzhou Yousheng Biotech Co., Ltd., Guangzhou, Guangdong 510060 People’s Republic of China; 5 Department of Oral and Maxillofacial Surgery, Guanghua School of Stomatology, Hospital of Stomatology, Institute of Stomatological Research, Sun Yat-sen University, Guangdong Provincial Key Laboratory of Stomatology, Guangzhou, Guangdong Province 510055 People’s Republic of China

**Keywords:** XPR1, TSCC, NF-κB signaling, Prognostic marker, Therapeutic target

## Abstract

**Background:**

Xenotropic and polytropic retrovirus receptor 1 (XPR1), a previously identified cellular receptor for several murine leukemia viruses, plays a role in many pathophysiological processes. However, the role of XPR1 in human cancers has not yet been characterized.

**Methods:**

Real-time PCR and western blotting assay were used to measure the expression of XPR1 in tongue squamous cell carcinoma (TSCC) tissues. Expression of XPR1 and p65 in clinical specimens was analyzed using immunohistochemical assay. The function of XPR1 on progression of TSCC was explored using in vitro and in vivo experiments. The molecular mechanism by which XPR1 helps to cancer progression was investigated by luciferase reporter activity, ELISA, PKA activity assay, immunofluorescence, western blotting and qPCR assay.

**Results:**

Herein, we find that XPR1 is markedly upregulated in TSCC tissues compared to normal tongue tissues. High expression of XPR1 significantly correlates with the malignant features and poor patient survival in TSCC. Ectopic expression of XPR1 increases, while silencing of XPR1 reduces the proliferation, invasion and anti-apoptosis capacities of TSCC cells. Importantly, silencing of XPR1 effectively inhibits the tumorigenecity of TSCC cells. Moreover, we identified that XPR1 increased the concentration of intracellular cAMP and activated PKA. Thus, XPR1 promoted phosphorylation and activation of NF-κB signaling, which is required for XPR1-mediated oncogenic roles and significantly correlates with XPR1 expression in clinical specimens.

**Conclusions:**

These findings uncover a critical role of XPR1 in TSCC progression via activation of NF-κB, and suggest that XPR1 might be a potential prognostic marker or therapeutic target.

**Electronic supplementary material:**

The online version of this article (10.1186/s13046-019-1155-6) contains supplementary material, which is available to authorized users.

## Background

Oral squamous cell carcinoma (OSCC) is one of the most common malignant tumors in the head and neck, and it is well-known for its highly proliferative and invasive nature. Tongue squamous cell carcinoma (TSCC) is the most common type of OSCC [1, 2]. Extensive surgery combined with chemotherapy and radiotherapy has been used as the primary clinical treatments for OSCC patients [3–5]. Despite considerable advances in diagnostics and therapies for this disease, the overall 5-year survival rate for OSCC patients stuck around 50% without marked improvement, because of the high risks of developing secondary or recurrent tumors in surrounding area [6–9]. Hence, this underscores the need for identifying the key molecular alterations that contribute to OSCC progression in order to identify effective therapeutic targets for patients.

Xenotropic and polytropic retrovirus receptor (XPR1) is a 696-amino acid protein with multiple transmembrane-spanning domains. It was first identified as a specific cell-surface receptor for xenotropic and polytropic classes of murine leukemia viruses (MLV) [10–12]. XPR1 has also been reported to mediate G protein recruitment and play a role in G-protein-coupled signal transduction [13–15]. Based on its homology to proteins involved in the regulation of phosphate transport, including PHO1, PHO90, and PHO91 [16–18], XPR1 has also been identified as an inorganic phosphate exporter in human cells [19].

Notably, recent studies suggest that there is a close relationship between levels of XPR1 expression and certain pathophysiological processes. For instance, Sharmaet et al. reported that activation of the receptor-activated NF-κB ligand (RANKL)-receptor activated NF-κB (RANK) signaling pathway in murine osteoclasts leads to XPR1 upregulation, which may suggest that XPR1 plays a role during bone resorption. This group also detected a differential distribution of XPR1, which is present in the cytoplasm of mononuclear osteoclast precursors and translocates to the membranes of mature multinucleated osteoclasts, suggesting that XPR1 has a role in differentiation in these cells [20]. Several studies have found that XPR1, which is demonstrated to interact with Beta-type platelet-derived growth factor receptor (PDGFRB) and play a fundamental role in the maintenance of the cellular phosphate balance in the brain, is a pathogenic gene involved in primary familial brain calcification (PFBC) [21–23]. Research by Hueso found that XPR1, a regulator of macrophage development, is upregulated in human atherosclerotic plaques during disease progression [24]. However, the clinical significance and biological roles of XPR1 in cancer progression remain largely unknown.

In the current study, we report that XPR1 expression is substantially increased in TSCC tissues and positively correlates with the clinical malignant features of TSCC as well as poor patient prognosis. Overexpression of XPR1 might promote TSCC cell proliferation, invasion and resistance to chemotherapy both in vitro and in vivo. Moreover, we show that XPR1 activates the NF-κB signaling pathway via upregulation of cAMP and subsequent PKA activation in TSCC. Collectively, our findings suggest that XPR1 plays a critical oncogenic role in TSCC progression via activation of NF-κB signaling, providing potential prognostic marker and therapeutic target against the disease.

### Patient specimen information

We conducted IHC staining on a total of 128 archived TSCC specimens, which were histopathologically diagnosed at the Sun Yat-sen University Cancer Center from 2001 to 2009. Patients’ consent and approval were obtained from the Institutional Research Ethics Committee prior to use. Information of specimens is summarized in Table S1. Eight freshly collected TSCC tissues and matched adjacent noncancerous tissues were stored in liquid nitrogen until use.

### Immunohistochemistry (IHC)

We performed IHC staining on the 128 paraffin-embedded TSCC tissue sections using anti-XPR1 (Sigma, HPA016557, 1:50) and anti-p65 (CST, #8242, 1:50) antibodies. In brief, paraffin-embedded specimens were cut into 4-μm sections and baked at 65 °C for 30 min. The sections were deparaffinized with xylenes and rehydrated. Sections were then submerged into EDTA antigenic retrieval buffer and microwaved for antigenic retrieval. Samples were treated with 3% hydrogen peroxide in methanol to quench the endogenous peroxidase activity, followed by incubation with 1% bovine serum albumin to block nonspecific binding, and then incubated with primary antibodies overnight at 4 °C. After washing, the tissue sections were treated with biotinylated anti-rabbit secondary antibody, followed by further incubation with streptavidin-horseradish peroxidase complex (Zsbio, BJ, China). Finally, the sections were immersed in 3-amino-9-ethyl carbazole and counterstained with 10% Mayer’s hematoxylin, dehydrated, and mounted in Crystal Mount. The XPR1 staining was graded with four scores, strong + 3, moderate + 2, weak + 1, and negative 0. Specimens with scores + 3, + 2 were defined as high expression; while the others scored as + 1 or 0 were low expression. On the other hand, specimens with > 10% nuclear p65 expression were defined as nuclear p65-positive, and specimens with ≤10% nuclear p65 expression were p65-cytoplasmic.

### Vectors, retroviral infection, and transfection

PCR-amplified human XPR1 coding sequence was cloned into the pLVX-IRES-puro vector. To silence endogenous XPR1, two RNAi oligonucleotides were cloned into the pSuper-retro-puro vector. shRNA sequences were as followed: shRNA#1: GCGAUUUGUGUGGAACUUCUU; shRNA#2: CGUGACACUAAGGUAUUGAUA. Transfection was performed using the Lipofectamine 2000 reagent (Invitrogen, Carlsbad, CA, USA) according to the manufacturer’s instructions. Stable cell lines expressing XPR1 or XPR1-shRNAs were selected for 10 days with 0.5 μg/ml puromycin. The cell lysates prepared from the pooled population of cells in sample buffer were fractionated using SDS-PAGE to quantify XPR1 protein levels.

### Western blotting analysis

Western blotting was performed using anti-XPR1 (Sigma, Saint Louis, MO, USA), anti-p65, and anti-p84 antibodies (Cell Signaling, Danvers, MA, USA). The membranes were stripped and re-probed with an anti-α-tubulin antibody (Sigma, Saint Louis, MO, USA) as a loading control.

### Colony formation assay

One thousand cells were plated in 6-well plate and cultured for 10 days. The colonies were fixed stained with 10% formaldehyde for 5 min and stained with 1.0% crystal violet for 30 s. Colonie numbers were counted and shown as mean + SD.

### Transwell penetration assay

1 × 10^4^ Cells were plated into the upper chamber of polycarbonate filters coated with Matrigel (BD Biosciences, San Jose, CA, USA) and cultured at 37 °C for 24 h. Invading cells were fixed in 1% paraformaldehyde, stained with crystal violet and counted in five random fields per well. The data was shown as mean + SD.

### Annexin V-FITC/propidium iodide (PI)-stained assay

Cells were trypsinized, washed in ice-cold PBS and centrifuged at 1000 g for 5 min. The pellet was resuspended in binding buffer at a density of 1.0 x l0^6^ cells/ml. Following this, 100 μl of the sample solution was incubated with 5 μl of FITC-conjugated Annexin V and 5 μl of PI for 15 min at 37 °C in dark. Subsequently, 400 μl of binding buffer was added to each sample and the samples were analyzed by flow cytometry. Apoptotic cells was caculated by the portion of Annexin V-positive cells.

### Xenograft tumor model

All animal experimental procedures were approved by the Institutional Animal Care and Use Committee of Sun Yat-sen University Cancer Center. Briefly, BALB/c-nu mice (5–6 weeks, 18–20 g, *n* = 6) were inoculated subcutaneously with 5 × 10^6^ SCC25 vector or XPR1-knockdown cells in the dorsal flanks. Tumor volumes were determined every week. Tumor volume was calculated using the eq. (L*W^2^)/2. After 7 weeks, the mice were euthanized and imaged, and the tumors were excised and weighed. Serial 6.0-μm sections were cut and subjected to immunohistochemical staining of Ki67 and TUNEL. Proliferation index was quantified by counting the proportion of Ki67-positive cells, while apoptotic index was quantified by the percentage of TUNEL-staining cells.

### Luciferase reporter assays

Cells (5 × 10^4^) were seeded in triplicate in 24-well plates and cultured for 24 h. One hundred nanograms of luciferase reporters plus 5 ng of pRL-TK Renilla plasmid (Promega, Madison, WI) were transfected into the indicated cells using Lipofectamine 2000 reagent (Invitrogen Co., Carlsbad, CA) according to the manufacturer’s recommendations. Luciferase and Renilla signals were measured 36 h after transfection using the Dual Luciferase Reporter Assay Kit (Promega, Madison, WI).

### ELISA analysis of cAMP and PKA activity assay

The concentration of cAMP and activity of PKA in the cell lysates of TSCC cell lines were measured using a cAMP Assay Kit (Abcam, ab65355) and PKA Kinase Activity Assay Kit (Abcam, ab139435) respectively, according to the manufacturer’s protocol.

### Immunofluorescence

Cells (5 × 10^4^) were plated on coverslips to culture for 24 h. Cells were washed three times with PBS and treated with 1% Triton X-100. Next, cells were stained with anti-p65 antibody (Cell Signaling Technology, 1:200) for 2 h at 4 °C. After washing three times with PBS, the cells were incubated with FITC-conjugated goat anti-mouse antibody (Cell Signaling Technology, 1:100) at 37 °C for 1 h. Cells were counterstained with DAPI (Sigma-Aldrich) to visualize the nuclei.

### Chemical reagents

NF-κB inhibitor quinazoline (QNZ) was purchased from Selleck Chemicals and used at a concentration of 10 nM.

### Statistical analysis

Statistical tests for data analysis included the log-rank test, χ2 test and Student’s two-tailed *t*-test. Multivariate statistical analysis was performed using a Cox regression model. Statistical analyses were performed using the SPSS11.0 statistical software package. Data represent the mean ± SD. *P* values of 0.05 or less were considered statistically significant.

## Results

### XPR1 overexpression correlates with progression and poor prognosis in TSCC

To investigate the role of XPR1 in cancer, we first analyze its expression. Interestingly, analysis with the Cancer Genome Atlas (TCGA) Head and Neck Cancer (HNSCC) dataset suggested that XPR1 was robustly increased in tumor samples compared to normal tissues (Additional file 1: Figure S1A). More importantly, high expression of XPR1 significantly predicted poorer overall survival in patients with HNSC in TCGA data, as indicated by the human protein atlas program (https://www.proteinatlas.org/, Additional file 1: Figure S1B). These data suggest that XPR1 might play an important role in the progression of head and neck cancer.

**Fig. 1:**
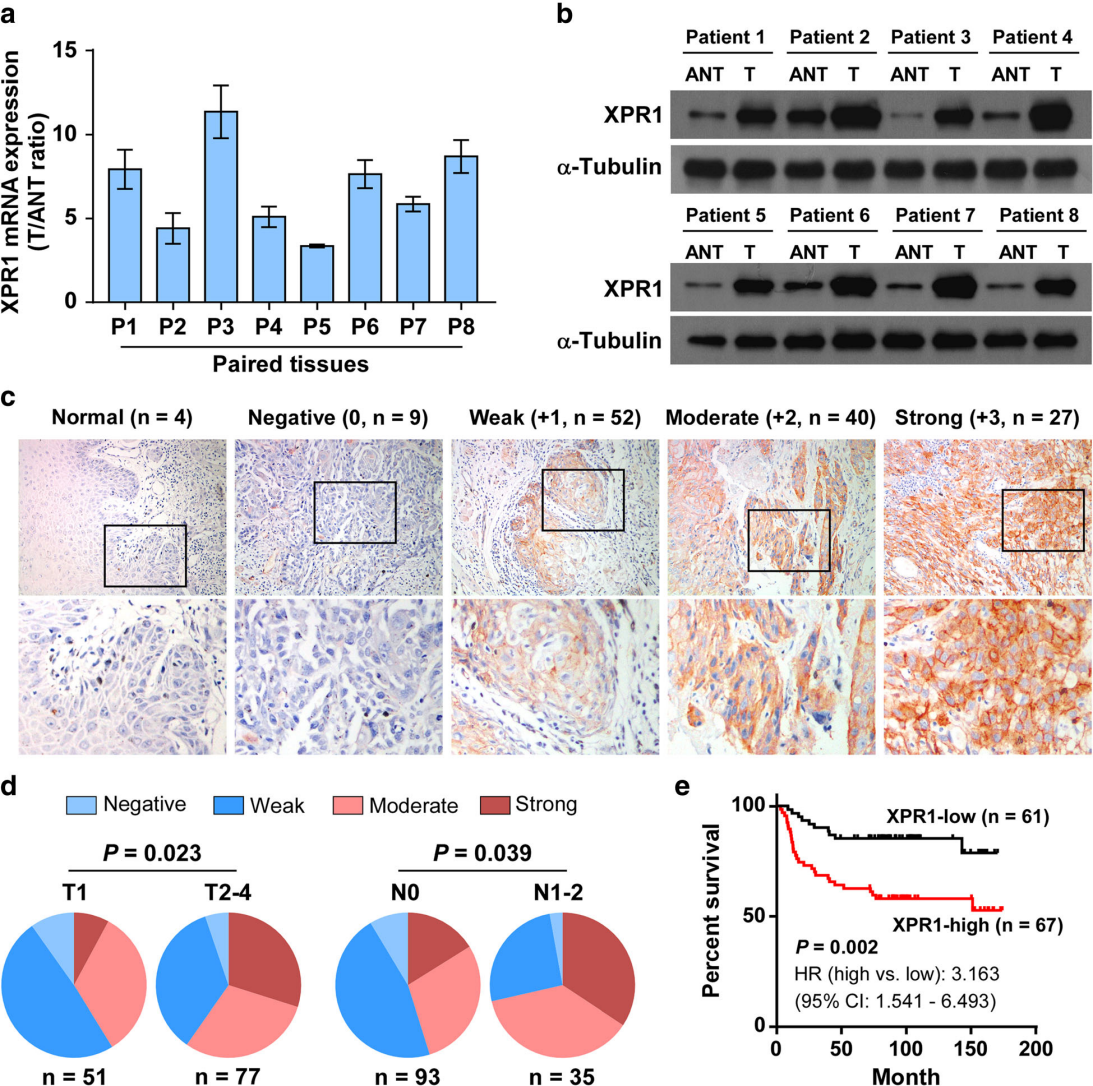
XPR1 overexpression correlates with progression and poor prognosis in TSCC. (**a**) Real-time PCR analysis of XPR1 mRNA in 8 paired human TSCC tissues (T) and the matched adjacent noncancerous tissues (ANT). Expression was normalized to GAPDH. (**b**) Western blot analysis of XPR1 protein in 8 paired TSCC tissues and controls. α-Tubulin was used as a loading control. (**c**) Representative images of XPR1 staining in 4 normal tongue tissues and 128 TSCC patient specimens, which was scored as negative 0, weak + 1, moderate + 2 and strong + 3. The number of each staining score was indicated (brackets). (**d**) Distribution and comparation between XPR1 staining and T classification or N classification. χ2 test was used. (**e**) Kaplan–Meier overall survival curve for TSCC patients stratified by low and high XPR1 expression (*n* = 128, log-rank test). HR, hazard ratio

We then validated the upregulation of XPR1 in TSCC, a common subtype of head and neck cancer. As shown in Fig. 1a and b, both mRNA and protein levels of XPR1 were robustly upregulated in 8 paired human TSCC tissues (T) compared with the matched adjacent noncancerous tissues (ANT). Furthermore, we assessed the expression status of XPR1 by immunohistochemistry (IHC) staining in 128 archived TSCC specimens and 4 normal tongue tissues (Additional file 1: Table S1). XPR1 was undetected in 4 normal tongue tissues (Fig. 1c). IHC analysis revealed that 27 contained strong (+ 3), 40 contained moderate (+ 2), and 52 had weak (+ 1) expression levels of XPR1, while 9 negatively (0) expressed XPR1 (Fig. 1c). Correlation analysis showed that the distribution of XPR1 staining was positively and significantly associated with T (*P* = 0.023) and N (*P* = 0.039) classifications in TSCC patients (Fig. 1d). The specimens with score + 3, + 2 were defined as XPR1-high, while the others were XPR1-low (Additional file 1: Figure S1C). Importantly, Kaplan-Meier survival curves and log-rank tests revealed that patients with high expression of XPR1 significantly had higher risk and poorer overall survival (*P* = 0.002, hazard ratio (95% CI) = 3.163 (1.541–6.493), Fig. 1e). Moreover, multivariate Cox regression analysis indicated that high expression of XPR1, patient age and T classification were each recognized as independent prognostic factors for the overall survival in TSCC (Additional file 1: Table S2). These results suggest that overexpression of XPR1 might contribute to TSCC progression, leading to poor clinical outcome.

### XPR1 promotes aggressiveness of TSCC cells in vitro

**Fig. 2:**
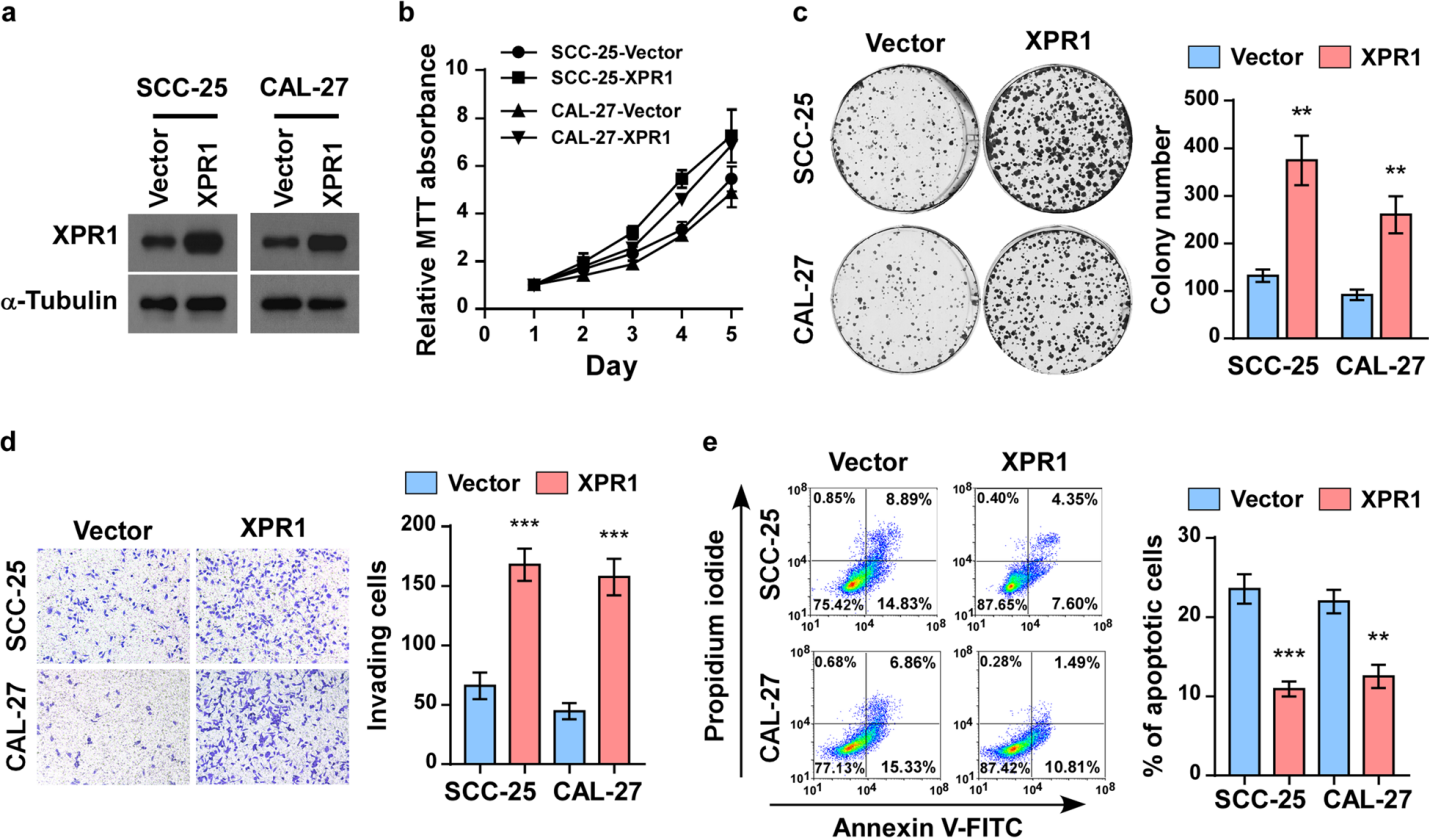
Overexpression of XPR1 promotes aggressiveness of TSCC cells in vitro. (**a**) TSCC cell lines SCC-25 and CAL-27 were stably transduced with XPR1. Overexpression of XPR1 was validated by western blot analysis. α-Tubulin was used as a loading control. (**b**) MTT assay of indicated cells. (**c**) Representative images and statastic analysis of colonies formed by indicated cells. (**d**) Representative micrographs and quantification of the invasiveness of XPR1-overexpressing cells in the transwell matrix invasion assay compared to vector control cells. (**e**) Flow cytometry analysis of annexin V-FITC/PI staining of the indicated cells treated with cisplatin (20 μM) for 24 h. **P < 0.01, ***P < 0.001

To investigate the biological role of XPR1, we first stably expressed XPR1 in TSCC cell lines SCC25 and CAL-27 by lentivirus infection (Fig. 2a). MTT assays revealed that ectopic expression of XPR1 promoted significant increase in viability of both SCC25 and CAL-27 cells (Fig. 2b), and these results were further confirmed by the colony formation assays (Fig. 2c). Moreover, transwell penetration assays showed that XPR1 strongly increased the invasive capacity of TSCC cells (Fig. 2d). In addition, XPR1 effectively reduced cisplatin-induced cell apoptosis, suggesting that XPR1 also contibuted to the resistance to chemotherapy in TSCC (Fig. 2e).

**Fig. 3:**
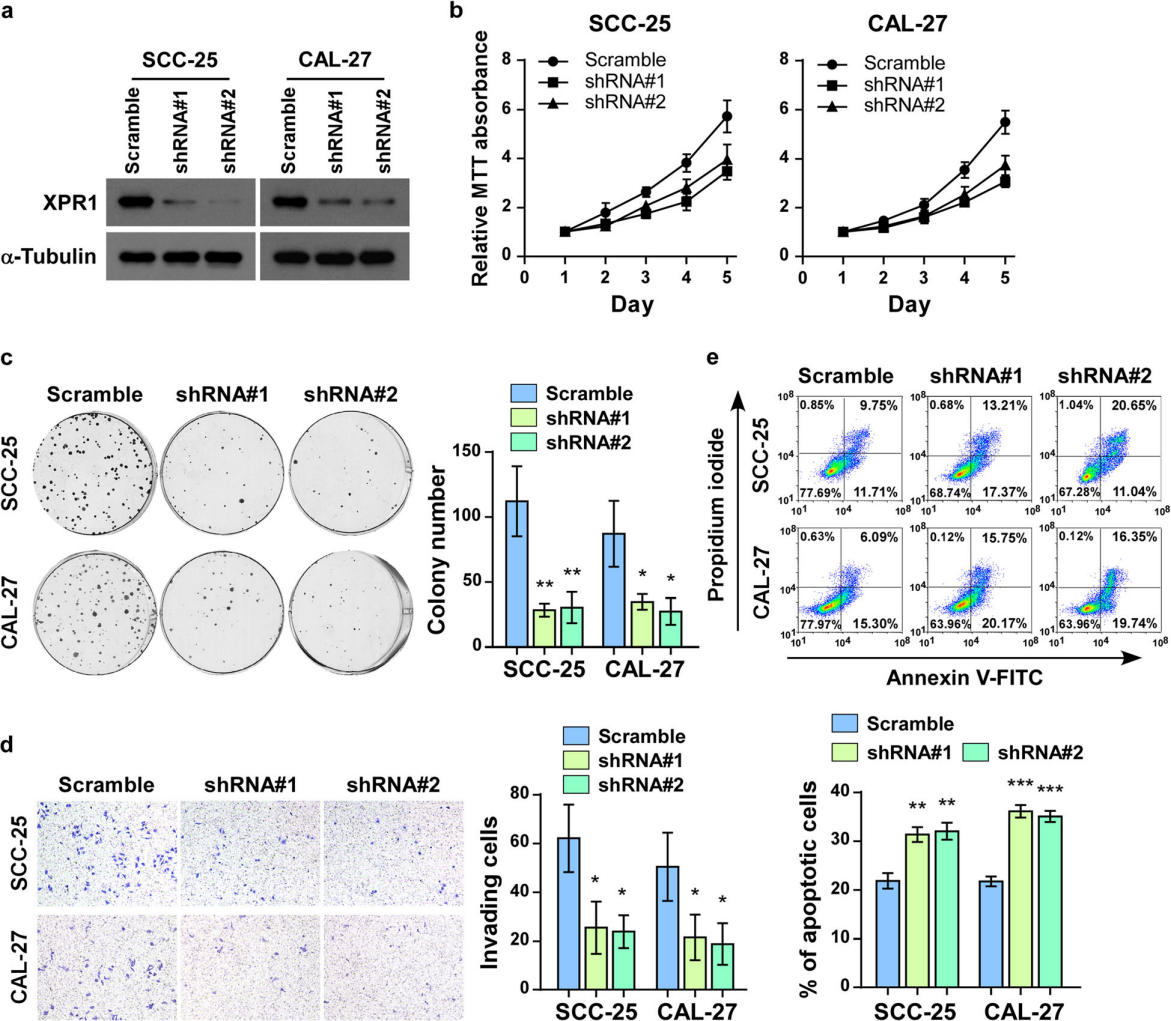
Silencing of XPR1 reduced TSCC aggressiveness in vitro. (**a**) TSCC cell lines SCC-25 and CAL-27 were stably transduced with two XPR1 shRNAs. Knockdown of XPR1 was validated by western blot analysis. α-Tubulin was used as a loading control. (**b**) MTT assay of indicated cells. (**c**) Representative images and statastic analysis of colonies formed by indicated cells. (**d**) Representative micrographs and quantification of the invasiveness of XPR1-silenced cells in the transwell matrix invasion assay compared to scramble control cells. (**e**) Flow cytometry analysis of annexin V-FITC/PI staining of the indicated cells treated with cisplatin (20 μM) for 24 h. Quantification was shown below. *P < 0.05, **P < 0.01, ***P < 0.001

On the other hand, silencing of XPR1 significantly reduced the proliferation, invasion and chemoresistance capacities of TSCC cells (Fig. 3a-e). Notably, alterations of XPR1 expression had no significant effects on basal apoptosis rate of TSCC cells (Additional file 1: Figure S2A and B). Collectively, these results suggest that XPR1 promotes aggressiveness of TSCC cells in vitro*.*

### Silencing of XPR1 inhibits tumorigenecity of TSCC cells in vivo

**Fig. 4:**
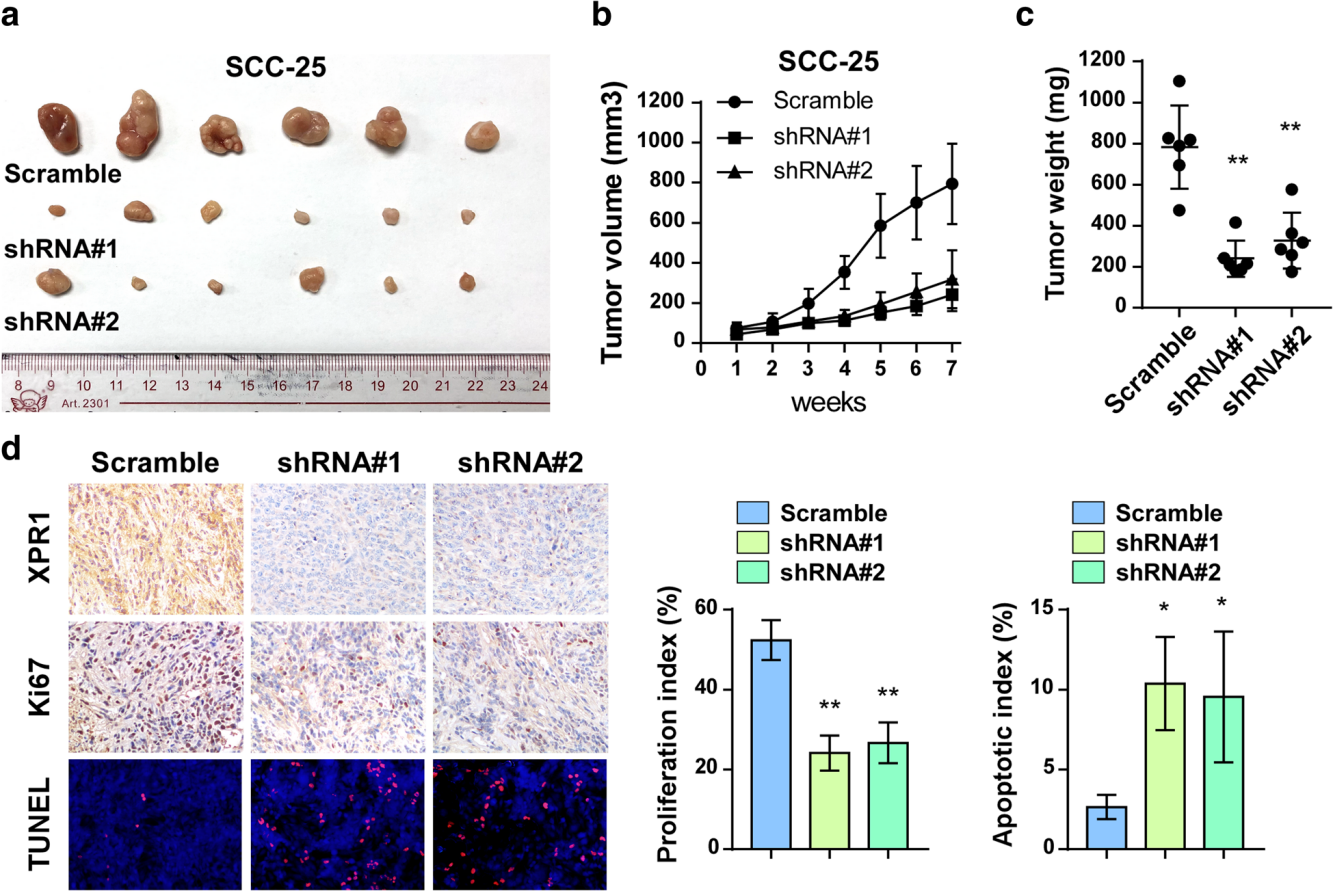
Silencing of XPR1 inhibits tumorigenecity of TSCC cells in vivo. (**a**) 5 × 10^6^ SCC-25 control and shRNAs-mediated XPR1 knockdown cells were subcutaneously injected into Balb/c nude mice and inocubated for 7 weeks. Tumors in each group were shown. (**b**) Tumor volumes were calculated weekly. (**c**) Tumor weights in each group. (**d**) IHC staining of XPR1 and Ki67, as well as TUNEL staining in xenograft section. Proliferation index was indicated by Ki67-positive cell percentage. Apoptotic index was calculated by the percentage of TUNEL-positive cells. *P < 0.05, **P < 0.01

We next investigated the role of XPR1 in the tumorigenicity of TSCC cells. 5 × 10^6^ control and shRNAs-mediated XPR1 knockdown SCC-25 were injected subcutaneously into Balb/c nude mice and inoculated for 7 weeks. Strikingly, tumors formed by XPR1 knockdown SCC25 cells grew much more slowly than scramble-transduced control SCC25 cells as indicated by the tumor volumes and weights (Fig. 4a-c). Consistently, IHC staining of Ki67 revealed that silencing of XPR1 inhibited cell proliferation of TSCC cells, but increased the percentage of TUNEL-stained cells in xenografts (Fig. 4d and e). These findings indicate that silencing of XPR1 reduced the tumorigenecity of TSCC cells, and suggest that XPR1 might be a therapuetic target.

### XPR1 activates NF-κB signaling

**Fig. 5:**
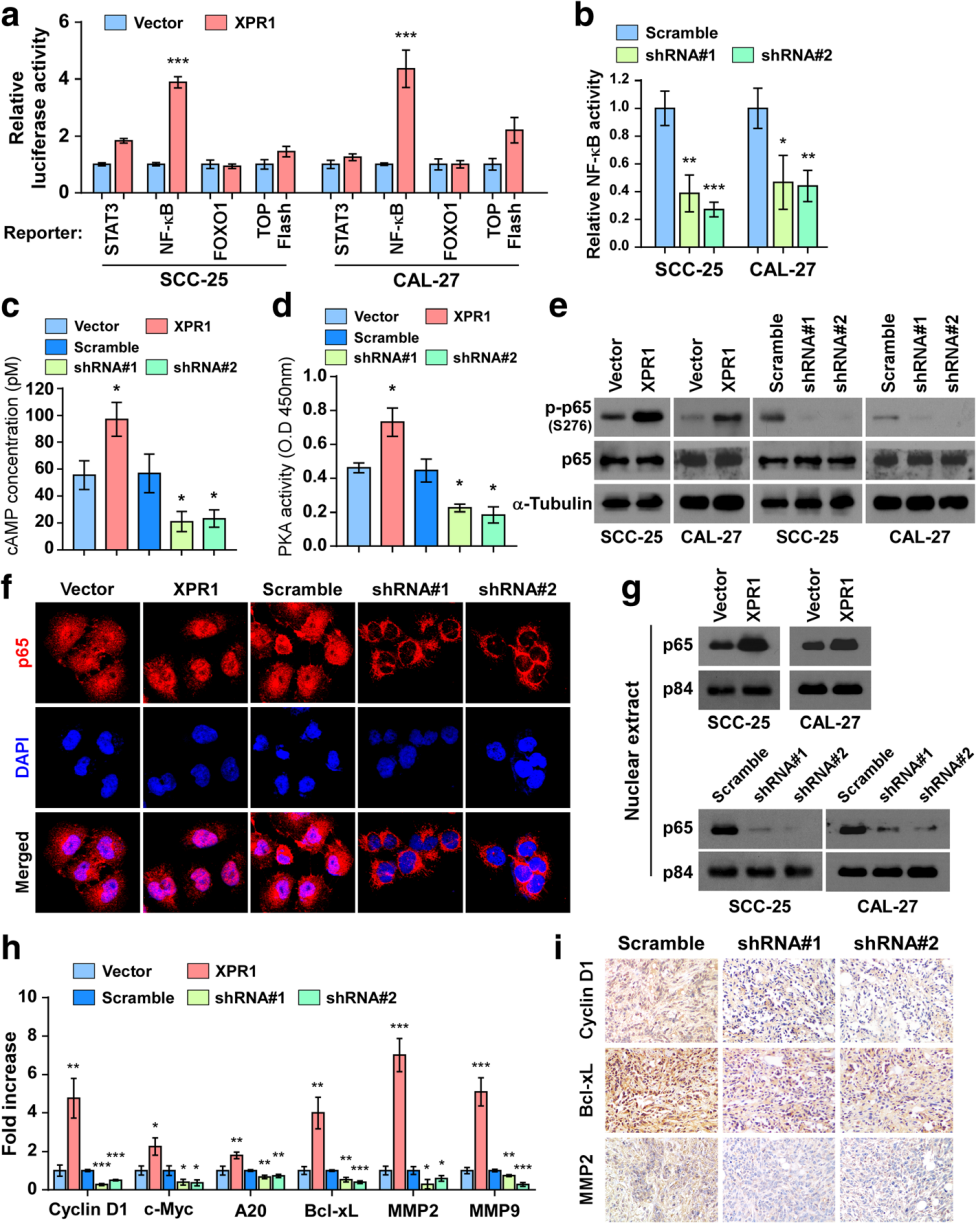
XPR1 activates NF-κB signaling. (**a**) Luciferase reporter assays of STAT3, NF-κB, FOXO1 and TOP Flash reporters. (**b**) Relative NF-κB activity in XPR1-silenced cells and controls. (**c**) ELISA analysis was performed to determine the intracellular cAMP concentration in the lystates of SCC-25 with altered expression of XPR1. (**d**) Activity of PKA in indicated cells was measured by absorbance at 405 nm. (**e**) Western blot analysis of p-p65-S276, p65 and α-Tubulin in indicated cells. (**f**) Fluorescence immunostaining of the p65 location in indicated cells. (**g**) Nuclear fractions were isolated, and p65 expression was examined by immunoblotting. p84 was used as a nuclear protein marker. (**h**) Relative expression of NF-κB downstream genes was analyzed by real-time PCR. (**i**) IHC staining of Cyclin D1, Bcl-xL and MMP2 in xenograft section. *P < 0.05, **P < 0.01, ***P < 0.001

To explore the mechanism for the oncogenic effects of XPR1 in TSCC, we conducted assays of luciferase reporters of serveral typical oncogenic pathways including STAT3, NF-κB, FOXO1 and Wnt/β-catenin. Interestingly, the luciferase reporter assays revealed that XPR1 markedly increased the activity of NF-κB, but showed little effects on other signaling pathways (Fig. 5a). Consistently, silencing of XPR1 significantly inhibited the luciferase activity of NF-κB in SCC-25 and CAL-27 cells (Fig. 5b). Notably, XPR1 was found as a G-protein-coupled receptor to stimulate cAMP production [1], and cAMP activates PKA kinase, which phosphorylates p65 at ser276 to promote nuclear translocation and transcriptional activation of NF-κB. Strikingly, our results indicated that overexpression of XPR1 increased, while silencing of XPR1 reduced the concentration of cAMP and activity of PKA in SCC-25 and CAL-27 cell lines (Fig. 5c and d). XPR1 increased the ser276 phosphorylation of p65 (Fig. 5e). Fluorescence immunostaining and cellular fractionation showed that overexpression of XPR1 promoted, while silencing of XPR1 inhibited the nuclear translocation of NF-κB/p65 in TSCC cells (Fig. 5f and g). Moreover, real-time PCR analysis revealed that XPR1 regulted the expression of typical NF-κB downstream genes (Fig. 5h), which was further confirmed by IHC staining in xenografts (Fig. 5i). Thus, our findings suggest that XPR1 activates NF-κB signaling by increasing cAMP levels and subsequent PKA-mediated phosphorylation of NF-κB.

### NF-κB activation was responsible for XPR1-mediated oncogenic effects

**Fig. 6:**
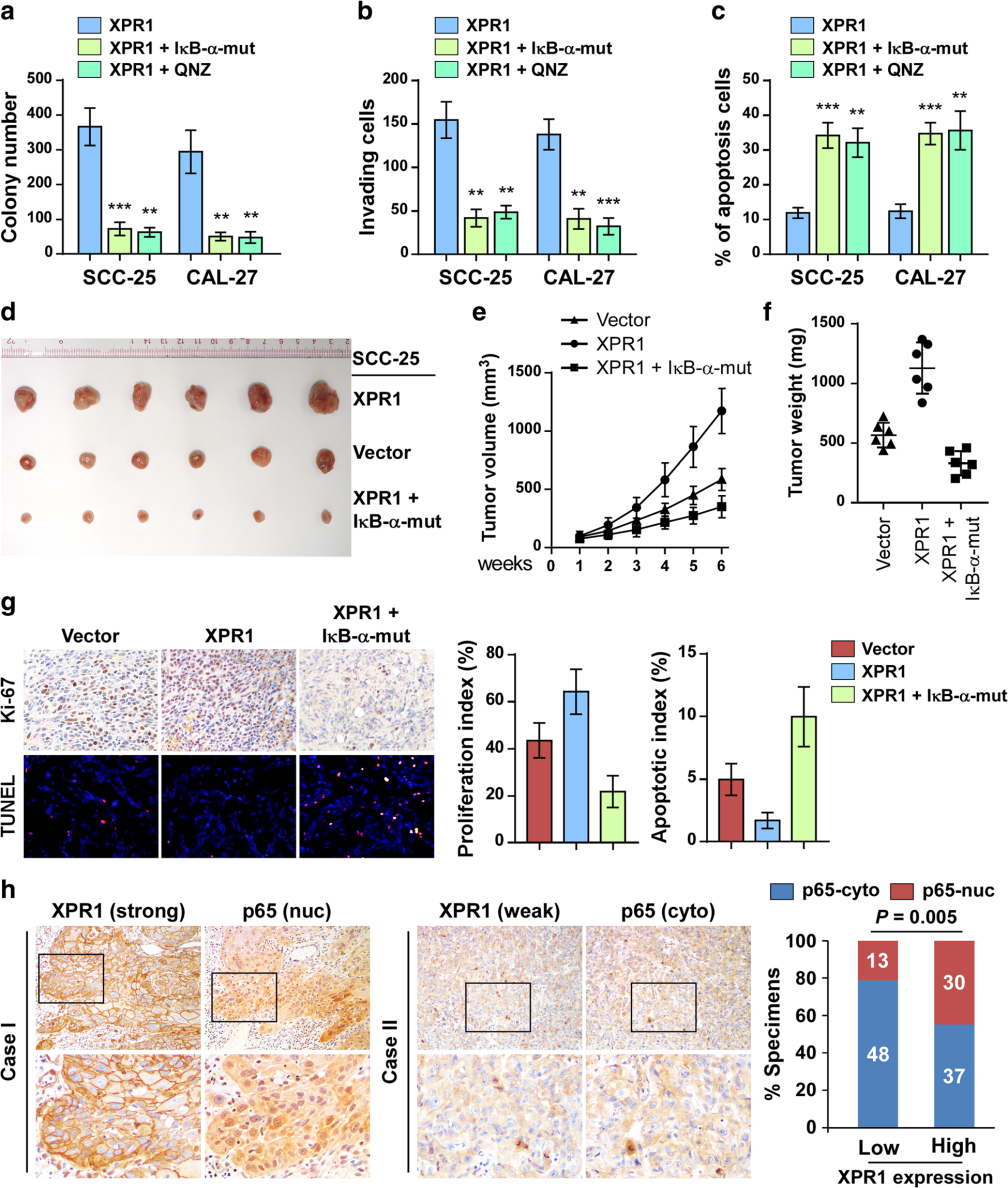
NF-κB activation mediates XPR1-mediated oncogenic effects. (**a**-**c**) XPR1-overexpressing SCC-25 and CAL-27 cells were transfected with IκB-α-mut or treated with NF-κB inhibitor QNZ at 10 nM for 24 h, and then applied for colony formation assay (**a**), transwell penetration assay (**b**), and annnexin V/PI assay (**c**). (**d**) 5 × 10^6^ vector control, XPR1-overexpressing, or XRP1 and IκB-α-mut-overexpressing SCC-25 cells were subcutaneously injected into Balb/c nude mice and inocubated for 6 weeks. Tumors in each group were shown. (**e**) Tumor volumes were calculated weekly. (**f**) Tumor weights in each group. (**g**) IHC staining of XPR1 and Ki67, as well as TUNEL staining in xenograft section. Proliferation index was indicated by Ki67-positive cell percentage. Apoptotic index was calculated by the percentage of TUNEL-positive cells. (**h**) Clinical relevace of XPR1 expression and NF-κB activation in patient specimens. Representative images of XPR1 and p65 IHC staining in 128 TSCC patient specimens. Correlation analysis revealed that high expression of XPR1 significantly associated with nuclear p65 expression. χ2 test was used. **P < 0.01, ***P < 0.001

We then investigated whether NF-κB activation was essential for the oncogenic effects by XPR1. We inhibited NF-κB activity in XPR1-overexpressing SCC25 and CAL-27 cells by transfection of IκB-α-mut plasmid which is non-degradative and restricts the p65/p50 complex in the cytoplasm, or treatment with NF-κB inhibitor QNZ (EVP4593) to potently reduced the phosphorylation of NF-κB and inhibit NF-κB transcriptional activation. As expected, both IκB-α-mut overexpression and QNZ administration inhibited NF-κB activity (Additional file 1: Figure S3A), leading to robust suppression of the proliferation, invadsion, and anti-apoptosis capacities of TSCC cells (Fig. 6a-c). Overexpression of IκB-α-mut significantly abrogated the promotive effects of XPR1 on the tumorigenecity of SCC-25 cells (Fig. 6d-f). Moreover, Ki67 IHC staining and TUNEL staining revealed that IκB-α-mut reduced the proliferation index and promoted cell apoptosis in vivo (Fig. 6g). Thus, these results suggest that NF-κB activation is essential for XPR1-mediated aggressiveness in TSCC.

### Clinical relevance of the XPR1/NF-κB axis in TSCC

Furthermore, we assessed whether the XPR1/NF-κB axis identified in our study was clinically relevant. IHC staining of p65 and XPR1 was performed in the same cohort of 128 TSCC patient specimens as indicated in Fig. 6h. We found that high expression of XPR1 significantly correlated with the nuclear expression of p65 (*P* = 0.005, Fig. 6h), which also predicted poor prognosis in TSCC patients (*P* = 0.010, hazard ratio (95% CI) = 2.224 (1.121–4.414), Additional file 1: Figure S3B).

Taken together, our findings suggest that overexpression of XPR1 activates NF-κB signaling, leading to the malignant progression and poor clinical outcomes in human TSCC.

## Discussion

XPR1, which was first identified as a cellular receptor for polytropic and xenotropic MLVs, has been shown to play a role in many pathophysiological processes. However, the clinical role of XPR1 in human cancers has not yet been characterized. Here, we demonstrate for the first time the potential role of XPR1 in TSCC progression and the potential value as a novel therapeutic target in human cancer. Clinical results show that high expression of XPR1 in TSCC patients correlated with a poor patient survival rate and more advanced stages. Overexpression of XPR1 induces TSCC cell aggressiveness with increased proliferation, invasion, and resistance to drug-induced apoptosis both in vitro and in vivo. Furthermore, XPR1 expression levels positively correlated with NF-κB signaling signatures, suggesting that XPR1 might be involved in regulation of the NF-κB signaling pathway in TSCC. Consistently, the significant correlation detected between XPR1 levels and NF-κB signaling hyperactivation was confirmed in a cohort of human TSCC samples. Hence, this study defines a critical mechanism for XPR1-mediated constitutive activation of NF-κB signaling, and identifies a new molecule with therapeutic potential for treating TSCC.

It has been acknowledged that the NF-κB pathway is an oncogenic signaling pathway that relevant to many aspects of tumorigenesis, including the control of apoptosis, cell cycle, invasion, differentiation, cell adhesion, cell migration, and angiogenesis [25]. To date, numerous researchers have confirmed that NF-κB signaling is constitutively activated in TSCC, and targeting NF-κB signaling contributes to characteristics of the malignant phenotype in TSCC, demonstrating the vital role of NF-κB signaling in TSCC progression. For instance, Lu KW et al. showed that Gyp, a component of Gynostemma pentaphyllum Makino, inhibits invasion and migration of human tongue SCC4 cells by downregulating related proteins RAS, NF-κB, COX2, ERK1/2, and MMP-9, finally reduces metastasis [26, 27]. Wang et al. have found that phosphorylated Ezrin (Tyr353) activates NF-κB, which results in enhanced metastasis of TSCC cells [28]. Activation of Ezrin and NF-κB is associated with cancer metastasis and poor patient prognosis [28]. Moreover, Su and colleagues showed that TRIM14, a member of the Tripartite Motif Containing (TRIM) family, was upregulated in TSCC and correlated with a more aggressive TSCC phenotype via activation of NF-κB signaling [29]. Collectively, dysregulation of NF-κB signaling has been well-documented in TSCC. Therefore, elucidating the underlying molecular mechanisms that regulate NF-κB signaling in TSCC could be important for the development of clinical TSCC therapies. Here, we show that overexpressing XPR1 enhanced NF-κB responsive luciferase activity and the expression of numerous well-characterized downstream genes of NF-κB signaling, whereas silencing of XPR1 had the opposite effects. This suggested that XPR1 might contribute to NF-κB activation in TSCC.

Acting as a G-protein-coupled receptor, XPR1 was found to promote cAMP production [15]. Consistently, we found that XPR1 increased the intracellular concentration of cAMP. Thus, XPR1 activated PKA to phosphorylate p63 at ser276 site, leading to nuclear translocation and transcriptional activation of NF-κB. These findings unveil a mechanism for XPR1-mediated NF-κB activation. Interestingly, Miguel et al. showed that the transcription of mouse *Xpr1* was significantly upregulated along with the NF-κB activator *Ikkβ*, whereas the NF-κB inhibitor *Iκbα* was downregulated during disease progression [20]. The opposite results were observed in the mouse model in which *CD40* expression was silenced [24]. This finding indicates that expression levels of XPR1 are consistent with the activation of NF-κB signaling. Furthermore, XPR1 has also been reported to be overexpressed in human atherosclerotic plaques and localized in the perivascular adipose tissue, suggesting its relationship with chronic inflammation. In this study, we found the high expression of XPR1 could promote the abnormal activation of the NF-κB pathway. However, the mechanism of XPR1 upregulation in TSCC needs to be explored further. According to Miguel’s group, non-canonical NF-κB signaling might be relevant to the regulation of XPR1. Combined with our research, there may be a positive feedback interaction occurring between XPR1 and the NF-κB pathway.

Inorganic phosphate (Pi), an essential nutrient to living organisms, is usually concentrated to promote malignant progression of tumors [30]. Accordingly, some inorganic phosphate transporters are upregulated in cancer and high serum Pi concentration correlates with morbidity and mortality of cancer [31]. Notably, recent evidence also indicates that a strong antiproliferative action of Pi in MDA-MB-231 cell line [32]. Moreover, high Pi, cooperate with chemo-drug could be extremely toxic to particular cell types [33–35]. These studies implicate that Pi homeostasis might play a key role for cancer malignant progression [35]. Interestingly, XPR1 was recent found to promote export of Pi. In the current study, our results revealed that overexpression of XPR1 significant inhibited the cytotoxic effects of cisplatin on TSCC cells. It thus could be postulated that XPR1 may reduce the Pi concentration to promote chemoresistance of TSCC cells; however, this hypothesis remains to be further investigated.

## Conclusion

In summary, this study reveals that the elevated expression of XPR1 plays an important role in TSCC progression via activation of NF-κB signaling. Our findings demonstrate for the first time a critical role for XPR1 in the progression of TSCC, and this work further elucidates the function of XPR1 in human disease. Understanding the precise roles of XPR1 in the pathogenesis and progression of TSCC and activation of the NF-κB signaling pathway will increase our knowledge of the biological basis of cancer, and might enable the development of novel therapeutic strategies against TSCC.

## Additional file

Additional file 1: **Table S1**. Clinicopathological characteristics of 128 patient samples. **Table S2**. Univariate and multivariate analysis of factors associated with overall survival in 128 TSCC patients. **Figure S1**. **(A)** The XPR1 mRNA expression of TSCC tissues compared to normal controls by analyzing data set from The Cancer Genome Atlas (TCGA). **(B)** High expression of XPR1 significantly predicted poorer overall survival in patients with HNSC in TCGA data, as indicated by the human protein atlas program (https://www.proteinatlas.org/). **(C)** Number of specimen in different XPR1 staining score. **Figure S2. (A and B)** Flow cytometry analysis of annexin V-FITC/PI staining to determine the basal apoptosis rate of SCC-25 and CAL-27 cells with or without XPR1 overexpression (A) or knockdown (B). **Figure S3**. **(A)** Relative NF-κB reporter assay in indicated cells. **(B)** Kaplan-Meier overall survival curve for patients with p65-cytoplasma versus p65-nuclear. (PDF 659 kb)

## Abbreviations

HNSC: Head and Neck Squamous Cell Carcinoma
MLV: Murine leukemia viruses
OSCC: Oral squamous cell carcinoma
PFBC: Primary familial brain calcification
TRIM: Tripartite Motif Containing
TSCC: Tongue squamous cell carcinoma
XPR1: Xenotropic and polytropic retrovirus receptor
