# Supplementary material for: Xenotropic and polytropic retrovirus receptor 1 (XPR1) promotes progression of tongue squamous cell carcinoma (TSCC) via activation of NF-κB signaling
Source: J Exp Clin Cancer Res. 2019 Apr 17;38:167. doi: 10.1186/s13046-019-1155-6 (PMC6469095; doi:10.1186/s13046-019-1155-6)
Supplement: Supplementary file 1 — Table S1. Clinicopathological characteristics of 128 patient samples. Table S2. Univariate and multivariate analysis of factors associated with overall survival in 128 TSCC patients. Figure S1. (A) The XPR1 mRNA expression of TSCC tissues compared to normal controls by analyzing data set from The Cancer Genome Atlas (TCGA). (B) High expression of XPR1 significantly predicted poorer overall survival in patients with HNSC in TCGA data, as indicated by the human protein atlas program (https://www.proteinatlas.org/). (C) Number of specimen in different XPR1 staining score. Figure S2. (A and B) Flow cytometry analysis of annexin V-FITC/PI staining to determine the basal apoptosis rate of SCC-25 and CAL-27 cells with or without XPR1 overexpression (A) or knockdown (B). Figure S3. (A) Relative NF-κB reporter assay in indicated cells. (B) Kaplan-Meier overall survival curve for patients with p65-cytoplasma versus p65-nuclear. (PDF 659 kb) [file 13046_2019_1155_MOESM1_ESM.pdf]

Xenotropic and Polytropic Retrovirus Receptor 1 (XPR1) promotes progression of tongue squamous cell carcinoma (TSCC) via activation of NF-κB signaling

Table S1. Clinicopathological characteristics of 128 patient samples

Parameters	Number of cases (%)
**Gender**	
Female	54 (42.2)
Male	74 (57.8)
**Age (years)**	
< 50	53 (41.4)
≥ 50	75 (58.6)
**T classification**	
T_1	51 (39.8)
T_2	65 (50.9)
T_3	9 (7.0)
T_4	3 (2.3)
**N classification**	
N_0	93 (72.7)
N_1	19 (14.8)
N_2	16 (12.5)
**vital status**	
Dead	39 (30.5)
Alive	89 (69.5)
**XRP1 expression**	
Negative (0)	9 (7.0)
Weak (+1)	52 (40.6)
Moderate (+2)	40 (31.3)
Strong (+3)	27 (21.1)

Table S2. Univariate and multivariate analysis of factors associated with overall survival in 128 TSCC patients.

Characteristics	Univariate analysis		Multivariate analysis	
	HR (95% CI)	*P* values	HR (95% CI)	*P* values
**XRP1 expression** (high)	3.163 (1.541-6.493)	0.002	2.230 (1.062-4.682)	0.034
**Age** (≥ 50 years)	2.341 (1.138-4.813)	0.021	2.695 (1.296-5.603)	0.008
**T stage** (T2-4)	2.222 (1.082-4.564)	0.030	1.629 (0.777-3.417)	0.196
**N stage** (N1-2)	3.920 (2.085-7.370)	< 0.001	3.578 (1.852-6.912)	< 0.001

HR, hazard ratio; CI, confidence interval.

Supplementary Figure 1

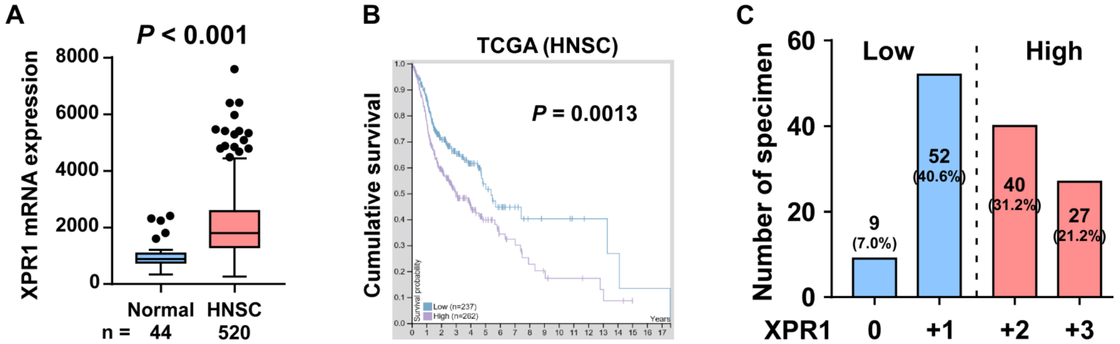

Figure S1. **(A)** The XPR1 mRNA expression of TSCC tissues compared to normal controls by analyzing data set from The Cancer Genome Atlas (TCGA). **(B)** High expression of XPR1 significantly predicted poorer overall survival in patients with HNSC in TCGA data, as indicated by the human protein atlas program (https://www.proteinatlas.org/). **(C)** Number of specimen in different XPR1 staining score.

Supplementary Figure 2

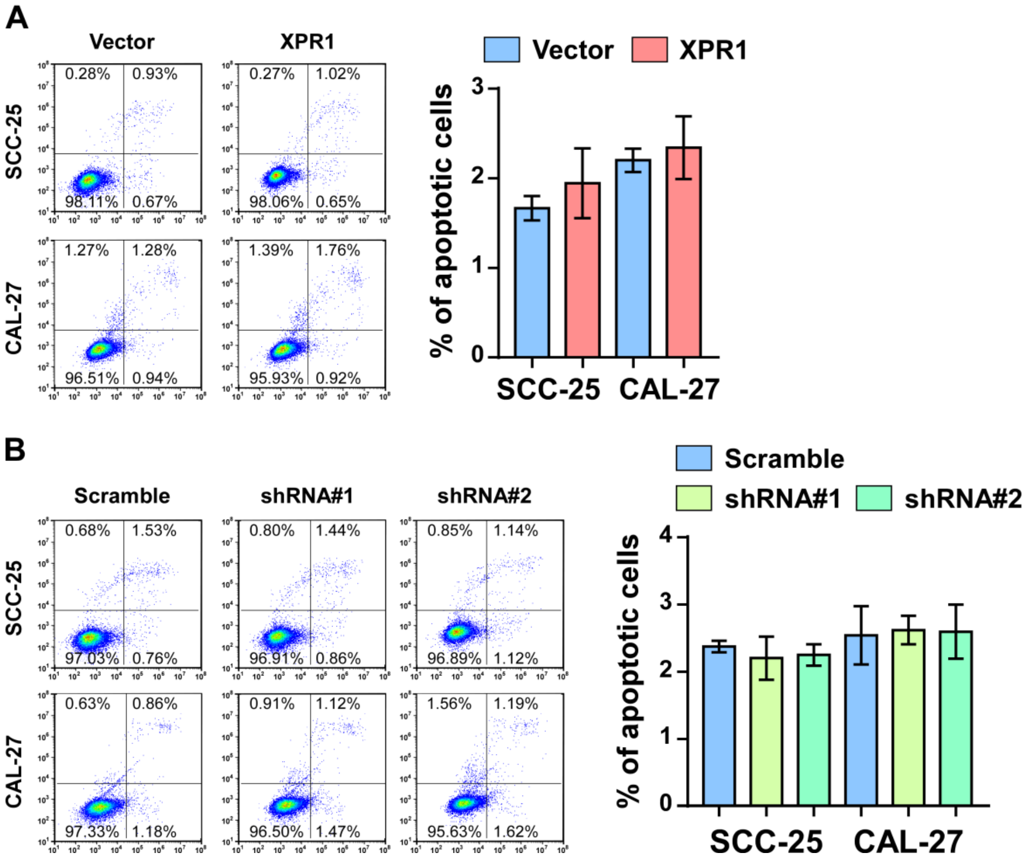

Figure S2. **(A and B)** Flow cytometry analysis of annexin V-FITC/PI staining to determine the basal apoptosis rate of SCC-25 and CAL-27 cells with or without XPR1 overexpression (A) or knockdown (B).

Supplementary Figure 3

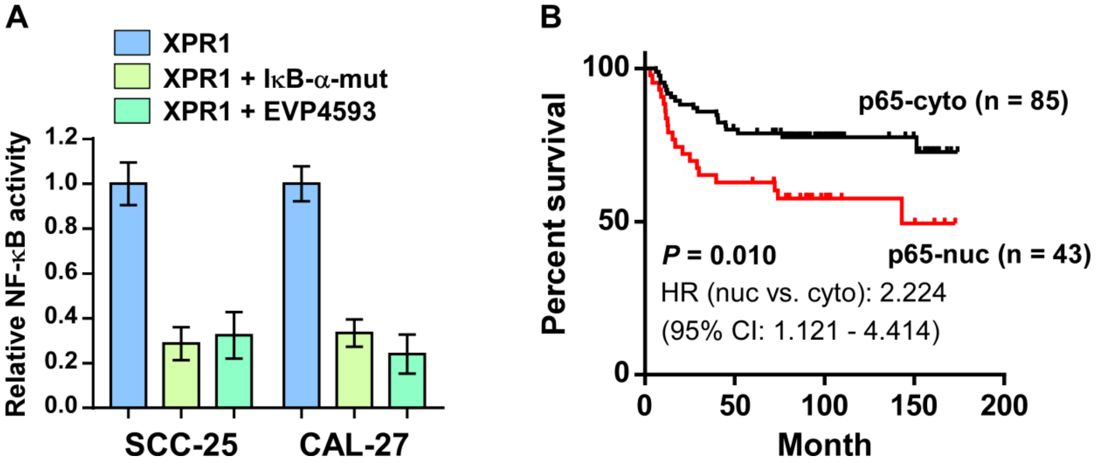

Figure S3. **(A)** Relative NF-κB reporter assay in indicated cells. **(B)** Kaplan-Meier overall survival curve for patients with p65-cytoplasma versus p65-nuclear.
